# Docking Based 3D-QSAR Study of Tricyclic Guanidine Analogues of Batzelladine K As Anti-Malarial Agents

**DOI:** 10.3389/fchem.2017.00036

**Published:** 2017-06-15

**Authors:** Nafees Ahmed, Sirajudheen Anwar, Thet Thet Htar

**Affiliations:** ^1^School of Pharmacy, Monash University MalaysiaPetaling Jaya, Malaysia; ^2^Department of Pharmacology and Toxicology, College of Clinical Pharmacy, Albaha UniversityAlbaha, Saudi Arabia

**Keywords:** batzelladine analogs, molecular docking, Discovery studio, anti-malarial, CDOCKER, 3D-QSAR

## Abstract

The *Plasmodium falciparum* Lactate Dehydrogenase enzyme (*Pf*LDH) catalyzes inter-conversion of pyruvate to lactate during glycolysis producing the energy required for parasitic growth. The *Pf*LDH has been studied as a potential molecular target for development of anti-malarial agents. In an attempt to find the potent inhibitor of *Pf*LDH, we have used Discovery studio to perform molecular docking in the active binding pocket of *Pf*LDH by CDOCKER, followed by three-dimensional quantitative structure-activity relationship (3D-QSAR) studies of tricyclic guanidine batzelladine compounds, which were previously synthesized in our laboratory. Docking studies showed that there is a very strong correlation between *in silico* and *in vitro* results. Based on docking results, a highly predictive 3D-QSAR model was developed with *q*^2^ of 0.516. The model has predicted *r*^2^ of 0.91 showing that predicted IC_50_ values are in good agreement with experimental IC_50_ values. The results obtained from this study revealed the developed model can be used to design new anti-malarial compounds based on tricyclic guanidine derivatives and to predict activities of new inhibitors.

## Introduction

Malarial infection caused by *Plasmodium falciparum* is the most deadly among all pathogens and challenge for developing countries (Bharate et al., 2007). Globally, ~3.3 billion people have been found at risk of affected by malaria in 2015, with sub-Saharan Africa population are at the highest risk of acquiring malaria. In 2014, 104 countries and territories have been considered as endemic to malaria (WHO, 2016 malaria report). Moreover, there has been rapid increase in resistance to drugs by *P. falciparum*. This resistance is considered because of emergence through mutation (Vennerstrom et al., 1999) or biochemical changes in the active site of drug targets (Foley and Tilley, 1998). The lactate dehydrogenase of *P. falciparum* (*Pf*LDH) has been used since long time as a potential molecular drug target for malaria (Penna-Coutinho et al., 2011). The LDH plays role in the inter-conversion of pyruvate to lactate in the glycolysis cycle, for energy production in living cells. Though chloroquine and other quinolone act primarily by inhibiting heme polymerization (Egan and Ncokazi, 2005), chloroquine interacts, in addition, specifically with *Pf*LDH in the NADH binding pocket, occupying a position similar to that of the adenyl ring cofactor. Therefore, chloroquine is a competitive inhibitor of *Pf*LDH and inhibits the propagation of parasitic growth (Menting et al., 1997; Read et al., 1999; Ncokazi and Egan, 2005).

**Figure 1:**
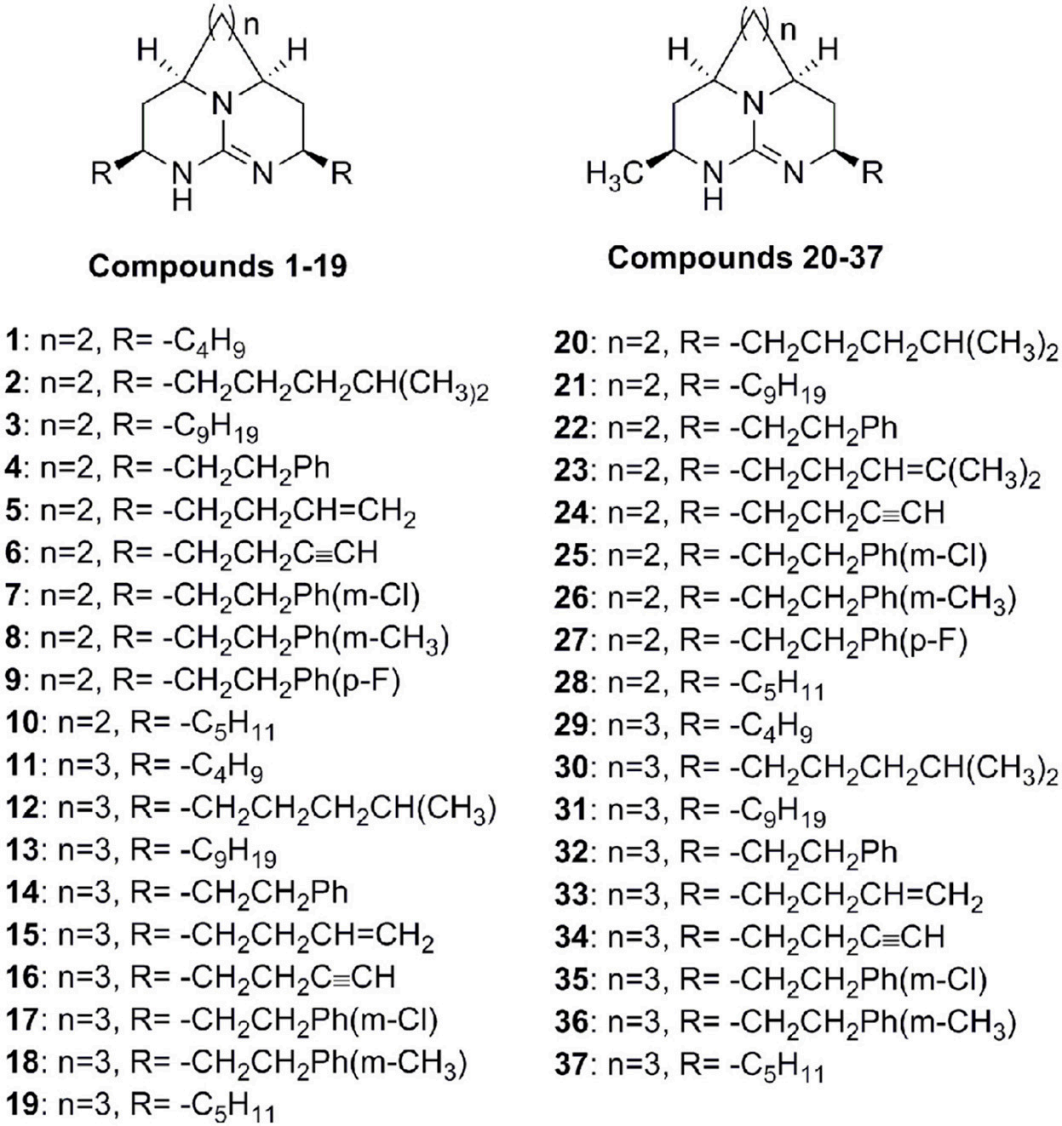
Structures of tricyclic guanidine compounds used in this study.

Recently, we have synthesized tricyclic guanidine batzelladine compounds (Figure 1) and reported the anti-malarial activity by using plasmodial LDH activity as measure of inhibition against various strains of *P. falciparum* that are resistant to chloroquine (Ahmed et al., 2013). We have obtained many potent inhibitors but none was as potent as chloroquine. Because of emergence of chloroquine resistant strain, it is an urgent demand to design and develop the new anti-malarial drug. In computer aided structure-based drug design, molecular docking is one of tools employed to study the putative geometry of a protein-ligand complex. In addition, docking is also used to predict binding affinities of ligands in virtual screening experiments. This help in rational-based synthesis of new compounds (Vieth and Cummins, 2000). In an attempt to develop the potent inhibitor of *Pf*LDH, in this paper, the molecular docking of tricyclic guanidine compounds and then 3D-QSAR model generation by using molecular field analysis have been performed and reported.

## Materials and methods

### Molecular docking

A set of 37 compounds (Figure 1), which covers most potent, moderate potent as well as least active, was selected from our previous work (Ahmed et al., 2013). These compounds were constructed by using ChemBiodraw ultra12. All compounds were then opened in Discovery studio suit 3.5 (Accelrys)^1^ and energy minimization was carried out by CHARMm (Rarey et al., 1996) force field using ligand partial charge method CFF (Consistent Force Field). Minimization was carried out until energy gradient of 0.01 was reached. The IC_50_ values of compounds were converted to pIC_50_ (−log IC_50_). The CDOCKER was used for docking of all compounds. In our previous work, we used LDH assay using chloroquine as a standard (Ahmed et al., 2013). The x-ray crystal structure of *Pf*LDH with chloroquine was retrieved from the PDB (1CET) (Read et al., 1999). The water molecules were deleted and hydrogen atoms were added. Finally protein was refined with CHARMm in DS 3.5 at physiological pH. To validate the docking reliability, co-crystalized ligand (chloroquine) was first re-docked to the binding site of *Pf*LDH enzyme. Consequently, all compounds were docked into same active site; twenty conformations of each compound were obtained through CDOCKER. The conformations with lowest energy were selected as the most probable binding conformation for each ligand used for 3D-QSAR studies.

### 3D-QSAR study

For 3D-QSAR study, the most preferred conformations of compounds were aligned together using a molecular overlay method (50% electrostatic and 50% steric fields). Total of 37 compounds were divided into training set (29 compounds) and test set (8 compounds). They were separated in two sets in order to have good molecular diversity in both groups. The 3D-QSAR (GridBasedTemp) model was generated using Discovery studio suit3.5. Two probe types are used to compute the energy grids that indicate electrostatic and steric effects. The CHARMm force field was used considering the electrostatic potential and the van der Waals potential as separate terms. A +1 charge is used as the electrostatic potential probe and distance-dependent dielectric constant is used to mimic the solvation effect. A carbon atom with 1.73 Å radius is served as a probe to measure the van der Waals potential. Rather than the full potential, a soft-core potential suggested in CDOCKER is used (Wu et al., 2003). A grid space of 1.5Å was kept with 5 as number of components. Full cross validated Partial Least-Squares (PLS) method of LOO (leave-one-out) was used to perform regression analysis (Golbraikh and Tropsha, 2000). The pIC50 values serve as dependent variable. To further validate the stability and predictive ability of this model, test set containing eight compounds was used.

## Results and discussion

### Molecular docking

**Figure 2:**
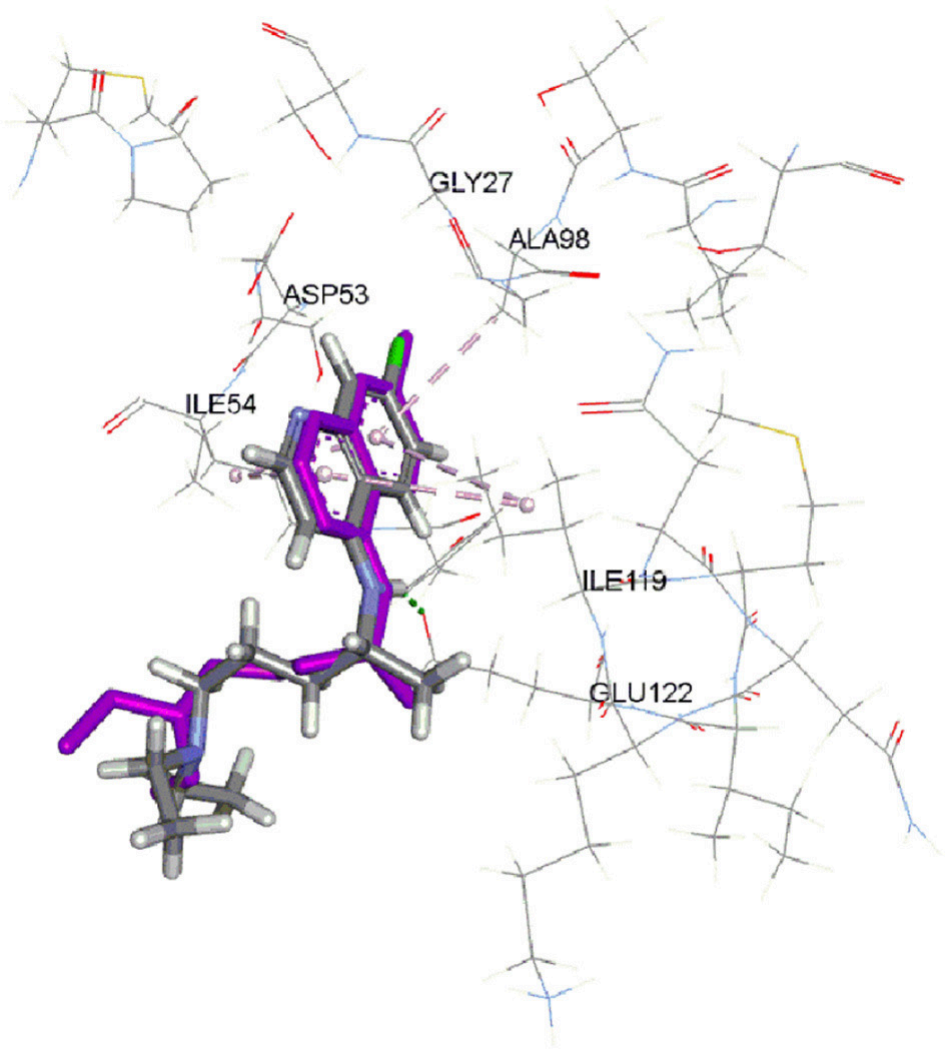
Docked chloroquine in the binding pocket of LDH enzyme. (Purple color is crystal structure pose of chloroquine from pdb ID: 1CET).

The CDOCKER is CHARMm-based docking algorithm that uses the CHARMm family of force fields and offers all the advantages of full ligand flexibility (including bonds, angles, and dihedrals) and reasonable computation times (Brooks et al., 1983; Shoichet and Kuntz, 1991; Akdogan et al., 2011). The CDOCKER algorithm involves a method that generate several initial ligand orientations in the active site of target protein. These poses further undergoes molecular dynamics based simulated annealing and final refinement by energy of poses were generated (Mo et al., 2012). The CDOCKER was used for docking of all compounds. In our previous work, we used LDH assay using chloroquine as a standard (Ahmed et al., 2013). There are two crystal structures of *Pf*LDH available in protein databank (pdb ID: 1CET and 2X8L). We have used 1CET as it has chloroquine bound to active site of enzyme. To validate the docking reliability, chloroquine was first re-docked to the binding site of *Pf*LDH enzyme (PDB ID: 1CET) and the docked conformation corresponding to lowest energy was chosen as the most probable binding conformation. As reported by Read et al. an aromatic ring of chloroquine forms van der Waals interactions with Ile54, Ala98, and Ile119. Moreover, carboxylate of Glu122 forms hydrogen bond with 4-NH group of chloroquine. This bulk of hydrophobic atoms of amino acids are important factor for orientation and binding of chloroquine (Read et al., 1999). The docked chloroquine in binding pocket of enzyme is shown in Figure 2.

The Root-mean-square deviation (RMSD) of docked chloroquine conformation was 1.03 Å, which suggested a high docking reliability of CDOCKER to reproduce the experimentally observed binding mode for *Pf*LDH inhibitors. Consequently, all compounds (Figure 1) were docked into same active site; 20 conformations of each compound were obtained. The conformations with lowest energy were selected as the most probable binding conformation for each ligand. The CDOCKER interaction energies of most favorable pose and IC_50_ values are tabulated in Table 1. The *r*^2^ of 0.75 was obtained between CDOCKER interaction energy and IC_50._

**Table 1:** CDOCKER interaction energies of most favorable pose and IC_50_ values of compounds.

**Compound**	**CDOCKER Interaction energy (-Kcal /mol)**	**IC_50_ (μM)^a^**
**1**	15.421	6.49
**2**	32.951	1.98
**3**	38.937	1.48
**4**	17.435	6.16
**5**	10.109	10.62
**6**	19.304	9.66
**7**	28.914	6.34
**8**	28.353	4.23
**9**	31.341	5.62
**10**	27.819	3.01
**11**	24.067	2.99
**12**	33.838	1.52
**13**	41.899	1.39
**14**	24.861	6.27
**15**	23.475	1.69
**16**	18.212	14.13
**17**	35.902	1.25
**18**	33.520	1.37
**19**	28.688	2.25
**20**	24.224	3.04
**21**	27.561	4.59
**22**	24.018	7.62
**23**	4.618	16.05
**24**	4.809	20.6
**25**	14.415	10.09
**26**	17.472	9.39
**27**	10.89	15.76
**28**	11.588	14.85
**29**	21.889	3.21
**30**	26.037	4.69
**31**	30.034	3.44
**32**	28.132	9.24
**33**	10.389	19.27
**34**	11.177	17.14
**35**	25.889	2.41
**36**	27.438	5.78
**37**	43.134	0.88

a *Ahmed et al., 2013*.

**Figure 3:**
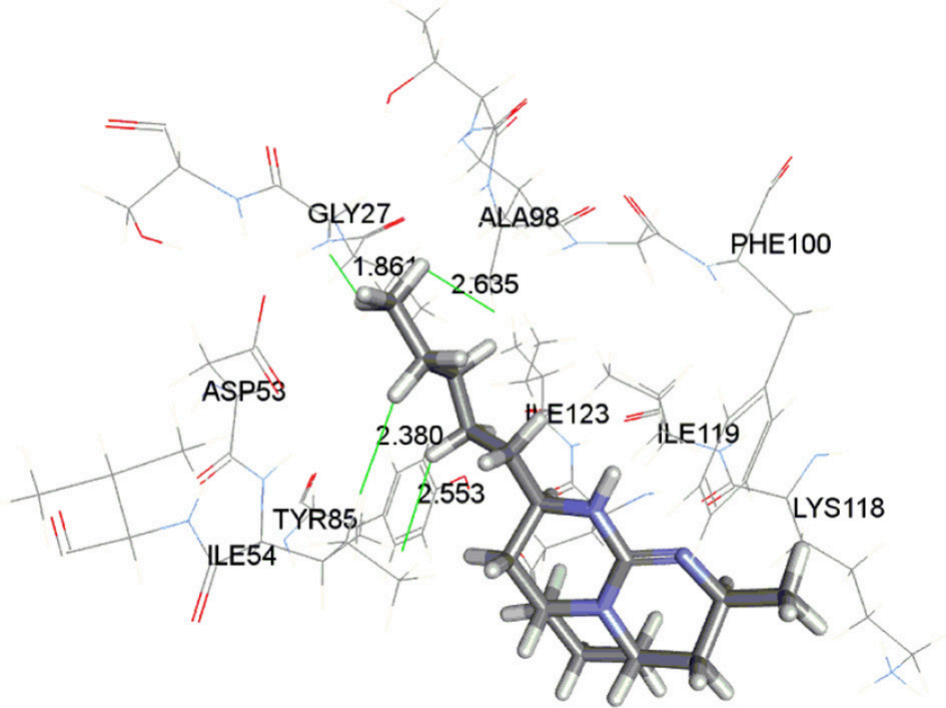
Binding of compound **37** in active site of enzyme LDH.

The docking study indicated that these compounds interact with Gly27, Ala98, and Ile54 residues via hydrophobic interaction, similar to that of chloroquine. The most active compound **37** is displayed in the Figure 3 indicating the hydrophobic interaction with Gly27, Asp53, Ile54, and Ala98 in the enzymatic binding pocket. Compound **37** was found to have lowest CDOCKER interaction energy (−43.25 kcal/mol); thus showing strong correlation between *in vitro* and *in silico* results. The conformations with lowest energy were selected as the most probable binding conformation for each ligand used for 3D-QSAR studies.

### 3D-QSAR study

**Figure 4:**
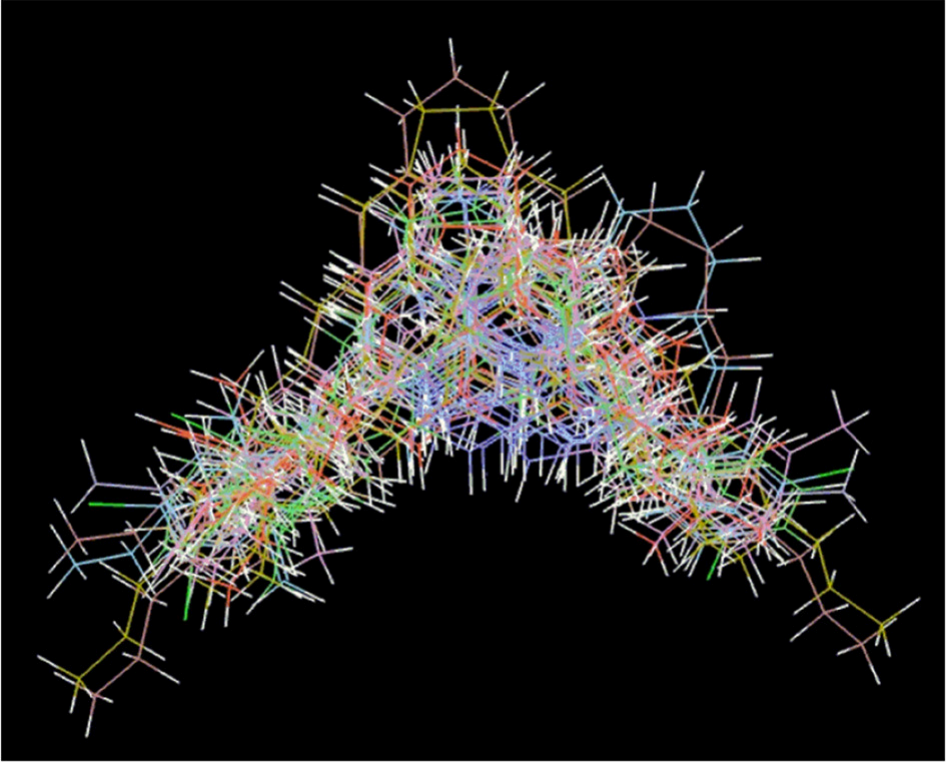
3D superimposed structures of compounds poses obtained from docking.

The most preferred conformations of compounds were aligned together using a molecular overlay method. The docked ligands poses were aligned as depicted in Figure 4. In a 3D-QSAR method, the 3D structures of a set of ligands are used to calculate energy potentials and then potential energy is used as descriptors to build a model. This model correlated the 3D structures and biological activities. 3D-QSAR model provides useful information about the correlation between the molecular fields and the activity (Verma et al., 2010). The predicted activity of a compound is a linear summation of the model coefficients multiplied by their corresponding grid values:

(1) $$\begin{array}{rcl} Activity\;\left(Predicted\right) & = & \;\overset{NEP}{\underset{i=1}{\sum }}CEP\left(i\right)VEP\left(i\right)\\ +\;\overset{NVDW}{\underset{i=1}{\sum }}CVDW\left(i\right)VVDW\left(i\right) \end{array}$$

where N_EP_ is the number of descriptors of electrostatic potential (EP), C_EP_(i) is the model coefficient for EP descriptor i, and V_EP_ is the electrostatic potential value on a grid point. Similarly, N_VDW_ is the number of descriptors of van der Waals (VDW) interaction, C_VDW_(i) is the model coefficient for VDW descriptor i, and V_VDW_ is the VDW interaction energy on a grid point.

**Figure 5:**
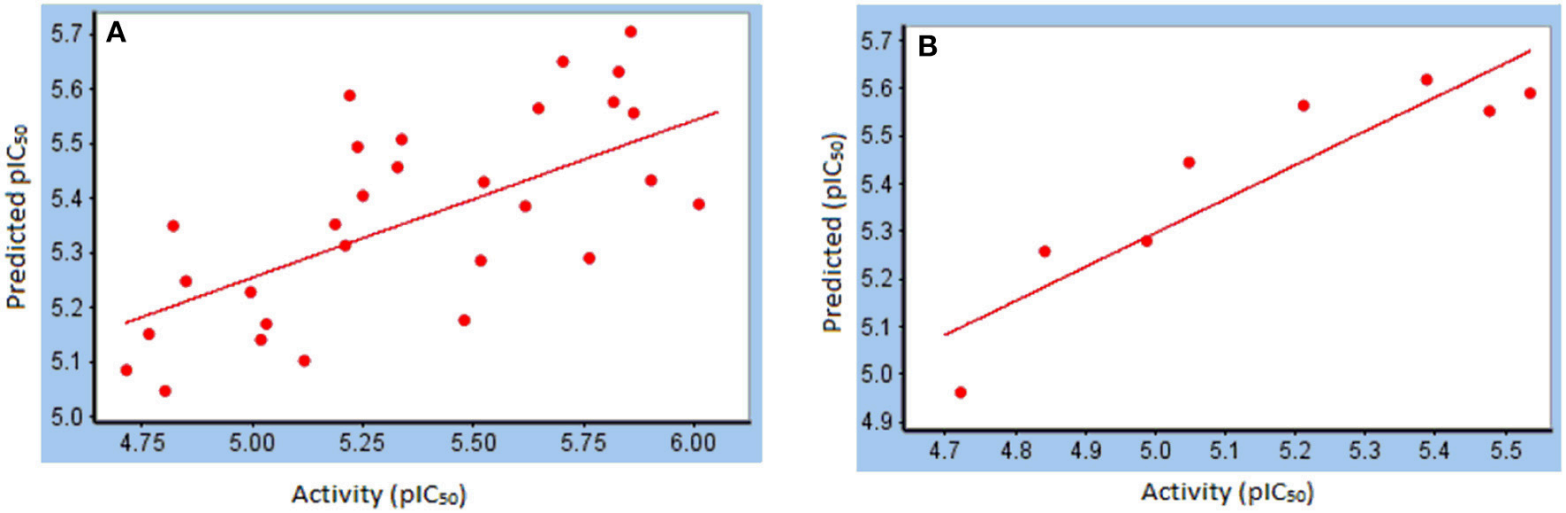
Correlation between experimental pIC_50_ and predicted pIC_50_ from **(A)** training set; **(B)** test set.

For training set, Partial Least Square analysis showed cross validated *q*^2^ of 0.516 and *r*^2^ of 0.81 with standard residual error of 0.18. Figure 5A indicate the correlation between the experimental and predictive pIC_50_ of compounds and results are listed in Table 2. The Leave-one-out (LOO) method of cross-validation was used for validation of predictive ability of model with the training set. Further, stability and predictive ability of this model was validated with test set. The predictive IC_50_ values are shown in Table 2. Figure 5B indicates the predictive values of pIC_50_ of test set by this model are in agreement with the experimental values with *r*^2^ value of 0.91.

**Table 2:** Experimental activities and predicted activities of compounds by 3D-QSAR model.

**Comp No**.	**pIC_50_**	**Predicted pIC_50_**	**Residual**	**Comp No**.	**pIC_50_**	**Predicted pIC_50_**	**Residual**
**1**	5.18	5.35	−0.17	**20**	5.51	5.30	0.21
**2**	5.70	5.77	−0.07	**21**	5.34	5.48	−0.14
**3**	5.83	5.87	−0.04	**22**	5.12	4.92	0.20
**4**	5.21	5.18	0.03	**23**	4.79	5.19	−0.40
**6**	5.01	5.02	−0.01	**25**	4.99	5.03	−0.04
**9**	5.25	5.30	−0.05	**26**	5.02	5.00	0.02
**11**	5.52	5.45	0.07	**27**	4.80	4.81	−0.01
**12**	5.81	5.76	0.05	**29**	5.49	5.08	0.41
**13**	5.85	5.95	−0.10	**30**	5.33	5.45	−0.12
**14**	5.20	5.55	−0.35	**33**	4.72	4.84	−0.12
**15**	5.77	5.36	0.41	**34**	4.77	4.89	−0.12
**16**	4.85	5.07	−0.22	**35**	5.61	5.51	0.10
**17**	5.90	5.78	0.12	**36**	5.24	5.45	−0.21
**18**	5.86	5.87	−0.01	**37**	6.05	5.47	0.58
**19**	5.65	5.65	0.00				
**TEST SET**							
**5**	4.97	5.29	−0.32	**24**	4.68	4.92	−0.24
**7**	5.19	5.57	−0.38	**28**	4.83	5.26	−0.43
**8**	5.37	5.62	−0.25	**31**	5.46	5.56	−0.10
**10**	5.52	5.60	−0.08	**32**	5.03	5.45	−0.42

**Figure 6:**
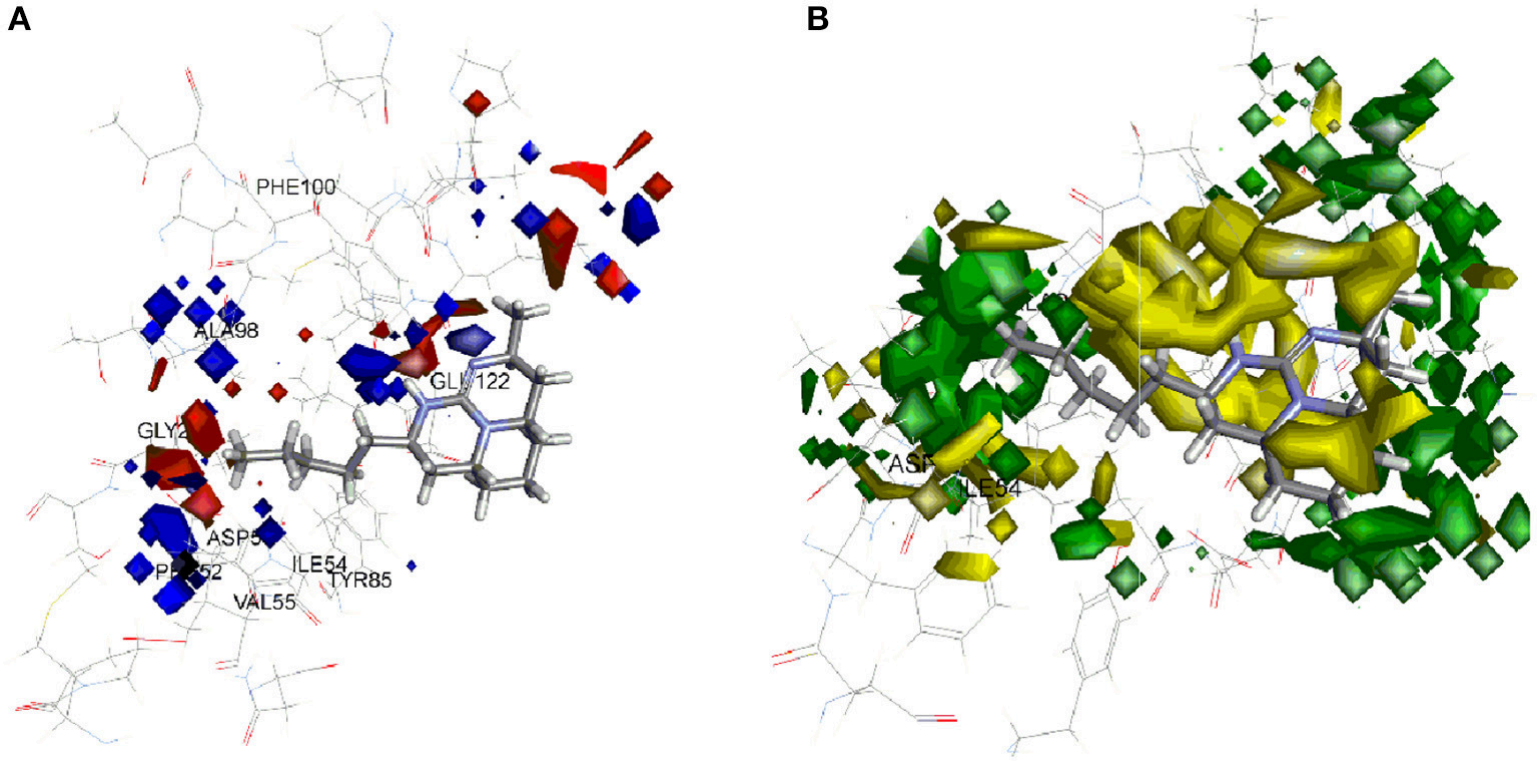
Molecular field analysis contour map displayed with compound **37** and superimposition on active site of *Pf*LDH **(A)** electrostatic interaction contour map; blue color represents positive coefficients while red color represents negative coefficients **(B)** steric interaction contour map; green color indicates positive coefficients and yellow color indicates negative coefficients.

The model coefficient of electrostatic potential is shown in Figure 6A. To aid visualization, the most active compound **37** is shown. Blue color represents positive coefficients while red color represents negative coefficients. To increase the activity (larger negative value of predicted activity), a molecule should have positive electrostatic potential in the blue area and negative in the red area. As depicted in Figure 6A, there is blue region below the tricyclic ring. In enzyme binding site, Glu122 is present near to this region which acts hydrogen bond acceptor forming hydrogen bond with hydrogen atom present on nitrogen of tricyclic ring, thus strengthen the binding and increase activity. Similarly, if polar group with positive potential (e.g., hydroxyl or phenol ring) is added on end of side chain of **37**, it will interact with Asp153 and strengthen inhibitory activity. The 3D view of steric interaction contour map is displayed in Figure 6B where green color indicates positive coefficients and yellow color indicates negative coefficients. To increase activity, a new molecule should have strong van der Waals attraction in the green area and weak van der Waals attraction in the yellow area. In other words, the new molecule should be designed in such a way that in the green area, the molecule shape should have a better match with the target system. More bulky substitution in the side chain of compound **37** improves the steric interaction. Compounds **7**, **8**, **17**, **26**, **35**, **36** containing aromatic ring in side chain showed good activity. In the enzyme binding pocket, this hydrophobic substituents interact with Gly27, Ala98, and Ile54 residues.

## Conclusion

We have developed the predictive GridBasedTemp model using docking based alignment for *Pf*LDH inhibitory activity of tricyclic guanidine compounds. The good model was obtained having leave-one-out cross validated *q*^2^ of 0.516 with maximum components of 5 having *r*^2^ value of 0.81 and 0.91 for training and test sets respectively suggesting the stability and robustness of model. This model is well matched with binding of most active compound **37** and chloroquine in the binding site of *Pf*LDH enzyme. Therefore, these results can be used to design new anti-malarial agents based on tricyclic guanidine derivatives and to predict activities of new inhibitors.

## Author contributions

NA outline the research strategy and idea. TH carried out the literature search. NA performed docking studies. SA drafted and revised the manuscript. All authors read and approved the final manuscript.

### Conflict of interest statement

The authors declare that the research was conducted in the absence of any commercial or financial relationships that could be construed as a potential conflict of interest.

## Footnotes

^1^Accelrys [http://accelrys.com/products/discovery-studio/]
